# Association of Combined Metals and PFAS with Cardiovascular Disease Risk

**DOI:** 10.3390/toxics11120979

**Published:** 2023-12-01

**Authors:** Yvonne S. Boafo, Sayed Mostafa, Emmanuel Obeng-Gyasi

**Affiliations:** 1 Department of Built Environment, North Carolina A&T State University, Greensboro, NC 27411, USA; 2 Environmental Health and Disease Laboratory, North Carolina A&T State University, Greensboro, NC 27411, USA; 3 Department of Mathematics and Statistics, North Carolina A&T State University, Greensboro, NC 27411, USA

**Keywords:** PFAS, metals, cardiovascular, mixtures, disease

## Abstract

This study sought to investigate the impact of exposure to metals and per- and polyfluoroalkyl substances (PFASs) on cardiovascular disease (CVD)-related risk. PFASs, including PFOA, PFOS, PFNA, and PFHxS, as well as metals such as lead (Pb), cadmium (Cd), and mercury (Hg), were analyzed to elucidate their combined effects on CVD risk. Methods: Utilizing data from the National Health and Nutrition Examination Survey (NHANES) spanning from 2007 to 2014, this investigation explored the effects of PFASs and metals on CVD risk. A spectrum of individual CVD markers, encompassing systolic blood pressure (SBP), diastolic blood pressure (DBP), high-density lipoprotein (HDL), low-density lipoprotein (LDL), total cholesterol, and triglycerides, was examined. Additionally, comprehensive CVD risk indices were evaluated, namely the Overall Cardiovascular Biomarkers Index (OCBI), including the Framingham Risk Score and an Overall Cardiovascular Index. Linear regression analysis was employed to probe the relationships between these variables. Furthermore, to assess dose–response relationships between exposure mixtures and CVD while mitigating the influence of multicollinearity and potential interaction effects, Bayesian Kernel Machine Regression (BKMR) was employed. Results: Our findings indicated that exposure to PFAS and metals in combination increased CVD risk, with combinations occurring with lead bringing forth the largest impact among many CVD-related markers. Conclusions: This study finds that combined exposure to metals and PFASs significantly elevates the likelihood of CVD risk. These results highlight the importance of understanding the complex interplay between multipollutant exposures and their potential implications for cardiovascular health.

## 1. Introduction

### 1.1. Cardiovascular Disease a Public Health Issue of Concern

Cardiovascular disease (CVD) affects millions of individuals worldwide and presents a significant public health challenge. The alarming increase in the prevalence of CVD, particularly in the context of social, environmental, and economic changes, calls for a comprehensive understanding of factors that increase CVD risk [1].

Despite CVD’s prevalence and far-reaching implications for healthcare and public health, the focus of clinical guidelines has predominantly revolved around addressing individual behavioral and dietary risk factors associated with CVD [2]. These factors typically include specific food choices, levels of physical activity, and tobacco and alcohol usage [3]. Unfortunately, environmental risk factors have been neglected in public health initiatives to prevent and manage cardiovascular disease [4].

Animal and human research suggests a potential connection between exposure to heavy metals and per- and polyfluorinated substances (PFASs), and an increased risk of cardiovascular disease [5,6]. However, very few studies have comprehensively assessed environmental exposure risk. Most human studies have adopted approaches involving single-contaminant exposure [7]. Indeed, few investigations have delved into the roles played by multiple metals and PFASs and their intricate interplay in shaping CVD risk [8].

Toxic elements, primarily metals, are released into the environment through various sources such as water, soil, and dust [9]. Furthermore, these contaminants’ persistence is such that they remain in the environment and human body long after exposure stops [10].

Human exposure to these toxic metals and PFASs occurs through various routes, including inhaling dust, the direct ingestion of contaminated soil and water, dermal contact with polluted soil and water, and the consumption of foods and tobacco products grown in fields contaminated with these elements [11,12]. The assessment of the specific amount of a particular metal entering the body can be determined through directly measuring its presence in human specimens, such as blood or urine, regardless of the exposure routes [13].

### 1.2. Metals Adversely Affect Cardiovascular Health

In the context of the US National Health and Examination Survey (NHANES), the biomonitoring of metals reveals a significant decrease in population-level exposure to certain heavy metals, notably lead (Pb) and cadmium (Cd) [14,15]. This reduction in metal exposure corresponds to a decrease in cardiovascular disease (CVD) mortality rates during the same period [16].

Numerous epidemiological studies have linked Pb exposure with CVDs such as peripheral arterial disease, coronary heart disease, and stroke [17,18,19]. The mechanistic pathway of how chronic Pb exposure causes CVD is through promoting oxidative stress, limiting nitric oxide availability, impairing nitric oxide signaling, augmenting adrenergic activity, increasing endothelin production, altering the renin–angiotensin system, raising vasoconstrictor prostaglandins, lowering vasodilator prostaglandins, and promoting inflammation [20]. In recent cross-sectional studies using data from the National Health and Nutrition Examination Survey (NHANES), Cd exposure measured in urine and blood has been associated with cardiovascular outcomes such as stroke and heart failure [21], myocardial infarction [22], peripheral arterial disease [23], and elevation in blood pressure [24]. Hg toxicity may be associated with hypertension and coronary heart disease in adults [25]. Recent studies have suggested that Hg is associated with cardiovascular dysfunction [26,27,28,29,30]. Specifically, Hg exposure has been associated with elevated BMI, a larger waist circumference, and higher diastolic blood pressure, total cholesterol, and triglycerides [31].

### 1.3. PFASs Adversely Affect Cardiovascular Health

Several observational studies have emphasized the association between exposure to PFASs and CVD risk and mortality [32]. The information for these studies came from population-based registries or longitudinal observational studies assessing the level of exposure with clinical events and control groups. When the clinical outcome was analyzed, excess arterial thrombosis was recognized in nearly all studies. Two possible explanations have been advocated: a worsening in cardiovascular risk or a direct prothrombotic activity of PFASs in exposed subjects. The effects of PFASs on cardiovascular risk factors, namely hyperlipidemia, diabetes mellitus, and arterial hypertension, have been investigated [33].

### 1.4. Mixed Exposures Better Model Environmental Effects on Cardiovascular Health

The combined effect of PFASs and metals on cardiovascular disease is understudied. Real-life human exposure to environmental stressors is highly variable and temporally dynamic at individual and population levels. Humans are constantly exposed to complex chemical mixtures of environmental contaminants such as Pb, Cd, Hg, and PFASs [34].

The objective of this study is to explore the effects of multipollutant mixtures of PFASs and metals on CVD risk. CVD risk will be operationalized using individual markers of CVD, an index developed in our previous research, and the Framingham Risk Score.

## 2. Materials and Methods

### 2.1. Sampling Strategy and Description Participants

NHANES is a multistage stratified survey constructed to give a comprehensive assessment of the nutritional and health status of a nationally representative sample of non-institutionalized persons in the US. The sampling process included sampling one county from each of 15 groups of counties. Then, sampling segments, households, and people were selected. This study uses four cycles of NHANES data for adults aged 18+ from 2007 to 2014. The study accounted for the complexities of the survey design using the survey package in R (version 4.2.1; R Foundation for Statistical Computing, Vienna, Austria).

### 2.2. Quantifying PFAS and Metals

The Centers for Disease Control and Prevention (CDC) measured 12 PFAS concentrations, including PFOA, PFOS, PFNA, and PFHxS, in the serum of NHANES participants aged 12 and older. The metal content of whole blood specimens was directly measured using mass spectrometry after a simple dilution sample preparation step (ICP-MS; CDC method No. ITB0001A). Detailed laboratory procedures are available via the CDC [35].

### 2.3. Measuring Cardiovascular Variables

Blood pressure was assessed with the individual seated comfortably, following a minimum rest period of five minutes. This was performed using a mercury sphygmomanometer in strict accordance with the established blood pressure measurement guidelines endorsed by the American Heart Association.

Lipid biomarkers were analyzed using the Beckman Synchron LX20 and Beckman UniCel^®^ DxC800 Synchron instruments at Collaborative Laboratory Services in Brea, CA, USA. The Roche Modular P chemistry analyzer was also utilized at the University of Minnesota in Minneapolis, MN, USA. The calculation of LDL cholesterol levels was performed using the Friedewald equation. Plasma levels of fasting and 2 h glucose were determined using a hexokinase assay carried out on a Roche/Hitachi 911 Analyzer and a Roche Modular P Chemistry Analyzer (Roche Diagnostics, Indianapolis, IN, USA).

#### 2.3.1. Cardiovascular Indexes

The Overall Cardiovascular Biomarkers Index (OCBI) was used as indicated by our prior work [36] and illustrated in Table 1 below. This index incorporates eight specific biomarkers, as suggested by the American Heart Association. Each biomarker was assigned an index value of either 0 or 1, in accordance with the cutoff values recommended by the American Heart Association and established clinical guidelines. A value of 0 was assigned to biomarkers with values deemed unacceptable. The OCBI was calculated via summing up the individual biomarker index values.

#### 2.3.2. Framingham Risk Score

To determine the Framingham Risk Score, we followed the risk scoring method established by Wilson et al. [37]. This approach involves assigning point scores to individuals based on categorical values of age, total cholesterol, high-density lipoprotein cholesterol, blood pressure, smoking status, and diabetes. The detailed scoring sheet are available in Wilson et al.’s study [37]. We calculated the risk score for each participant and determined the risk score based on sex. Criteria considered include age by gender, age and total cholesterol by gender, smoker by gender, HDL cholesterol by gender, whether the participant has systolic blood pressure, and whether they are treated or untreated. Use Appendix A to understand how points are located.

### 2.4. Statistical Analysis

#### 2.4.1. Descriptive Statistics

Descriptive statistics were calculated to describe the distribution of PFASs and metals, demographic variables, and CVD-related variables. Both Spearman and Pearson correlations were used to assess the relationships among the study variables. To test for differences between participants, the survey-weighted t-test was used for comparing means.

#### 2.4.2. Multivariable Linear Regression

The study used multivariable linear regression analysis to analyze the association between combined exposures to PFAS components PFOA, PFOS, PFNA, and PFHxS; the metals Hg, Pb, Cd; and individual CVD markers, the OCBI, and the Framingham Risk Score. CVD risk was the response variable in all the linear regression models. The models were fitted using the variables, and estimated coefficient and corresponding 95% confidence intervals were obtained. The fitted models were adjusted for age, BMI, and sex. The models were again adjusted for taking prescription medication for hypertension and whether the participants have been told to take cholesterol medication.

The linear regression model is mathematically represented as:

$$Y=\beta_{0\;}+\beta_{1\;}X_{1}+\beta_{2}\;X_{2}+\cdot \cdot \cdot +\beta_{p}X_{p}+\epsilon$$

such that $X_{j}$ denotes the jth predictor and $\beta_{j}$ estimates association between the predictors and the outcome variable. Residual plots were used to assess the validity of the linear regression assumptions, namely, linearity, homoscedasticity, and outliers. The assumptions were deemed satisfied after we removed some clear outliers from the data.

#### 2.4.3. Bayesian Kernel Machine Regression (BKMR)

The BKMR modeling approach was employed to evaluate the collective impact of PFOA, PFOS, PFNA, PFHxS, Hg, Pb, and Cd on variables associated with cardiovascular health. BKMR is a method that has been widely utilized by numerous researchers to evaluate the combined effect of multiple pollutants [1,2,3,4,5,6] on a wide range of health outcomes due to its capacity to model non-linear and non-additive relationships effectively. This is because the technique is adept at capturing complex interactions and dependencies within the data [38,39] and, furthermore, offers a more precise evaluation of the combined impact of contaminants on a specific outcome of interest.

The model employed in our study is as follows:

$$g\left(\mu_{i}\right)=h(z_{i1,}\ldots \ldots .,\;z_{iM})+{\beta X}_{i};\;i=1,\;\ldots .,\;n$$

In this model, g represents a monotonic link function, $\mu_{i}=E(Y_{i})$, h represents the flexible kernel function of exposures $z_{i1}\ldots \ldots .,\;z_{iM}$ with x being the vector of covariates with a linear association with CVD, and β represents a vector of associated coefficients [38].

In this study, the predictors, Z, are the PFAS and metal variables, and h(.) represents the exposure–response function. The BKMR utilizes a kernel function [38,39] to investigate the relationship between exposure and response, accounting for the intricate non-linear and non-additive connections between the PFAS components and CVD [38,39]. It employs the Markov chain Monte Carlo method for model fitting, posterior inclusion probability to identify the most significant exposures, and a hierarchical approach to identify crucial variables in a high-dimensional setting [6]. The BKMR model employs various representations to investigate the patterns of association and interaction between PFASs, metals, and CVD risk. This method provides insights into both univariate and bivariate relationships, interactions, and overall effects, as well as single-variable effects and interactions [39,40]. This method aids in addressing the intricate effects of exposure to environmental mixtures through considering various scenarios of exposure–response functions [38,39,40].

All analyses in this study were carried out using R (version 4.2.1; R Foundation for Statistical Computing, Vienna, Austria). The significance level was set at 0.05.

## 3. Results

### 3.1. Characteristics of the Sample Population

Table 2 summarizes the demographic attributes of the study’s total sample population of 6237 individuals. The mean age of the participants was 43.0 years, with a standard deviation (SD) of 5.23.

Gender distribution was nearly even, with 51.3% identifying as female. Educational attainment varied, with 30.41% of participants having attended some college or having attained an associate degree and 30.13% possessing a bachelor’s degree or higher. A smaller proportion of the sample had less than a high school education (18.10%) or held a high school diploma or its equivalent (21.35%).

Regarding race and ethnicity, most participants were of White ethnicity (67.75%), while smaller percentages were classified as Black (10.84%), Hispanic (14.16%), or belonging to other racial/ethnic groups (7.25%).

Household income exhibited diversity, with 42.53% of participants falling into the income bracket of USD 25,000–55,000, 31.72% earning less than USD 25,000, 19.72% having an income between USD 55,000 and USD 75,000, and 6.02% earning more than USD 75,000.

Marital status revealed that 62.8% of participants were either married or living with a partner, while 37.2% were single.

### 3.2. Distribution of Exposure and Study Variables

Table 3 displays summary statistics for environmental exposures and cardiovascular disease risk outcomes stratified by gender. The table shows the means and standard deviations (SD) for exposures including PFOA, PFOS, PFNA, PFHxS, Hg, Pb, and Cd and outcomes such as systolic and diastolic blood pressure, total cholesterol, triglycerides, LDL cholesterol, HDL cholesterol, OCBI, and Framingham Risk Score. The *p*-values indicate statistical significance.

**Figure 1:**
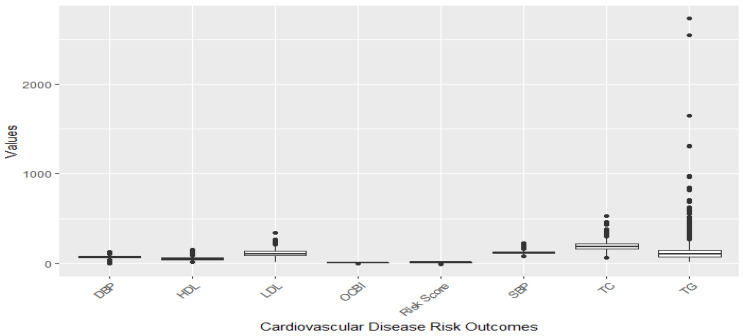
Distribution of CVD-related variables before omission of outliers.

**Figure 2:**
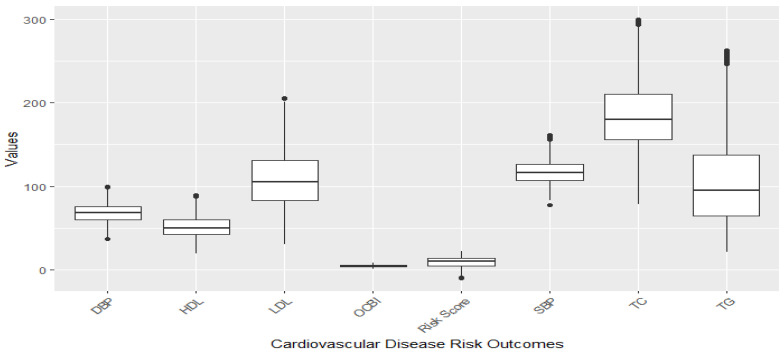
Distribution of CVD-related variables after omission of outliers with the 1.5xIQR rule.

Figure 1 and Figure 2 illustrate the distribution of individual cardiovascular markers in the study. The markers of focus encompass systolic blood pressure (SBP), diastolic blood pressure (DBP), Overall Cardiovascular Biomarker Index (OCBI), Framingham Risk Score (Risk Score), high-density lipoprotein cholesterol (HDL), low-density lipoprotein cholesterol (LDL), total cholesterol (TC), and triglycerides (TG).

Figure 1 illustrates a boxplot showing the distribution of the cardiovascular disease risk outcome before removing outliers, and Figure 2 shows the distribution after removing outliers. The study used a 1.5 threshold rule to multiply the interquartile range (IQR). Then, the product was subtracted from the lower boundary to determine the lower limit, and the product was added to the upper boundary to set the upper limit. Figure 1 shows significant outliers with TG, TC, and LDL. Figure 2 gives a better understanding of the distribution of these risk outcomes after removing the outliers.

### 3.3. Correlation between Pollution Variables and Cardiovascular-Related Variables

**Figure 3:**
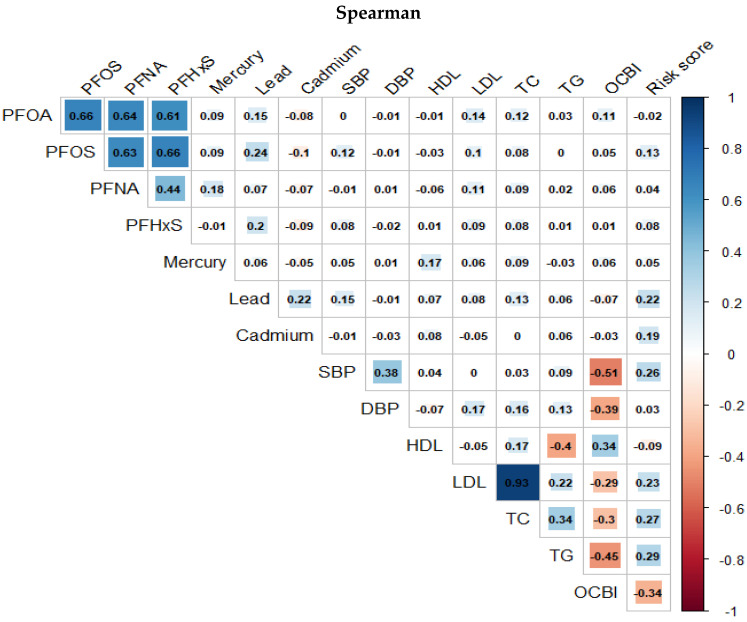
Spearman correlation between pairs of PFASs, metals, and cardiovascular-related variables.

**Figure 4:**
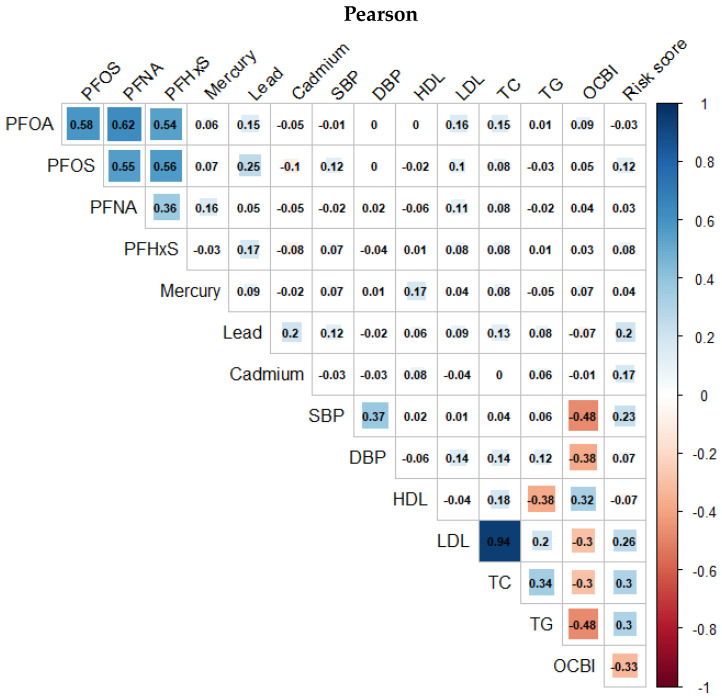
Pearson correlation between pairs of PFASs, metals, and cardiovascular-related variables.

Figure 3 and Figure 4 presents the Spearman and Pearson correlation analyses conducted on the exposure and outcome variables in the study, respectively. The overall trend indicates a strong monotonic correlation between PFAS variables, and a similarly high degree of correlation is observed among the cardiovascular-related variables from the Spearman correlation compared to a much more linear association shown by the Pearson correlation plots.

### 3.4. Association between Pollution Variables and Cardiovascular-Related Variables

Table 4A,B assess the association between PFAS and metal exposures on the one hand and systolic and diastolic blood pressure, LDL cholesterol, total cholesterol, HDL cholesterol, triglycerides, and the OCBI on the other hand. This assessment considers interactions and adjusts for various covariates, including age, BMI, gender, taking hypertension medication, and cholesterol prescription medication. The results present coefficient estimates with their 95% confidence intervals and associated *p*-values. The findings highlight significant associations and interactions between these exposures and CVD-related outcomes, shedding light on the impact of these exposures on cardiovascular disease risk. Specifically, an increase in cadmium is associated with an increase in systolic blood pressure. This is significant with a coefficient estimate of 8.72 (95% CI: 2.22 to 15.21, *p* = 0.0106). Additionally, an increase in age is associated with an increase in Systolic Blood Pressure. This is significant with coefficient estimate of 0.32 (95% CI:0.28, 0.36, *p* = 0.0001). While the result shows that being a male is associated with higher diastolic blood pressure, significant with a coefficient estimate of 3.37 (95% CI:1.04 to 3.69, *p* = 0.0010, the results show that being a male is associated with lower total cholesterol, with a coefficient estimate of −9.65 (95% CI:−13.12 to −6.19, *p* = 0.0001).

### 3.5. Association between Pollution Variables and Framingham Risk Score

In Table 5, we examined the association between exposure to PFASs and metals and the Framingham Risk Score, taking gender into account and adjusting for BMI.

### 3.6. BKMR Analysis

Bayesian Kernel Machine Regression (BKMR) analyses were conducted to investigate the relationships between environmental contaminants, specifically PFAS and metal exposures, and various CVD-related health outcomes. The results are summarized in Table 6, which provides the Posterior Inclusion Probability (PIP) for each contaminant in relation to different health variables. In the context of this analysis, the PIP quantifies the probability of each contaminant being included as a relevant factor in explaining the variation in the respective health outcome.

For instance, a PIP value of 0.9548 for PFOS in relation to diastolic blood pressure suggests a very high probability that PFOS is an influential factor affecting diastolic blood pressure levels. On the other hand, a PIP of 0.0790 for PFOA in relation to LDL cholesterol indicates a moderate likelihood that PFOA is a relevant factor in explaining variations in LDL cholesterol levels.

Table 7, Table 8, Table 9, Table 10, Table 11 and Table 12 provide the results of a hierarchical Bayesian Kernel Machine Regression (BKMR) analysis for DBP and SBP, total cholesterol, and HDL and LDL cholesterol. The analysis categorizes exposure variables into groups and presents Group PIP (Posterior Inclusion Probability) and Cond PIP (Conditional Inclusion Probability) values for each group.

For DBP, Group 1 includes the PFAS contaminants (PFOS, PFOA, PFHxS, PFNA), all of which have high Group PIP values (0.910), but only PFOS has a Cond PIP of 1.0000, suggesting a dominant influence on diastolic blood pressure. Group 2 comprises the heavy metals (Pb, Hg, Cd) with a high Group PIP value (0.994). Among these metals, lead has the highest Cond PIP value (0.0805), indicating a moderate impact on diastolic blood pressure.

For systolic blood pressure, Group 1 consists of the PFAS contaminants (PFOS, PFOA, PFHxS, PFNA). These PFAS substances have a low Group PIP value (0.050), with varying Cond PIP values. PFNA stands out with a relatively higher Cond PIP of 0.6800, suggesting a stronger impact on systolic blood pressure compared to the other PFASs. Group 2 represents the heavy metals (Pb, Hg, Cd) with a Group PIP value of 0.368. Lead has the highest Cond PIP (0.9945), indicating a substantial influence on systolic blood pressure.

For total cholesterol, Group 1 includes the PFAS contaminants (PFOS, PFOA, PFHxS, PFNA), all of which have a high Group PIP value (0.988), but only PFOS has a Cond PIP of 0.9089, suggesting a dominant influence on total cholesterol. Group 2 comprises the heavy metals (Pb, Hg, Cd) with high a Group PIP value (1.000). Among these metals, Pb has the highest Cond PIP value (1.0000), indicating a moderate impact on total cholesterol.

For DBP, these results suggest that while both PFASs and heavy metals are relevant, PFOS, mercury, and lead stand out as particularly important exposures in explaining variations in diastolic blood pressure. For systolic blood pressure, lead appears to have the most prominent impact on this health outcome, and for total cholesterol, PFOS and lead are important in explaining variation in total cholesterol.

For LDL cholesterol, these results suggest that while both PFASs and heavy metals are relevant, PFNA and lead stand out as particularly important exposures in explaining variations in diastolic blood pressure. For LDL cholesterol, lead and PFNA appear to have the most prominent impact on this health outcome and for LDL cholesterol.

#### 3.6.1. Univariate Association of PFASs and Metals with Systolic and Diastolic Blood Pressure

**Figure 5:**
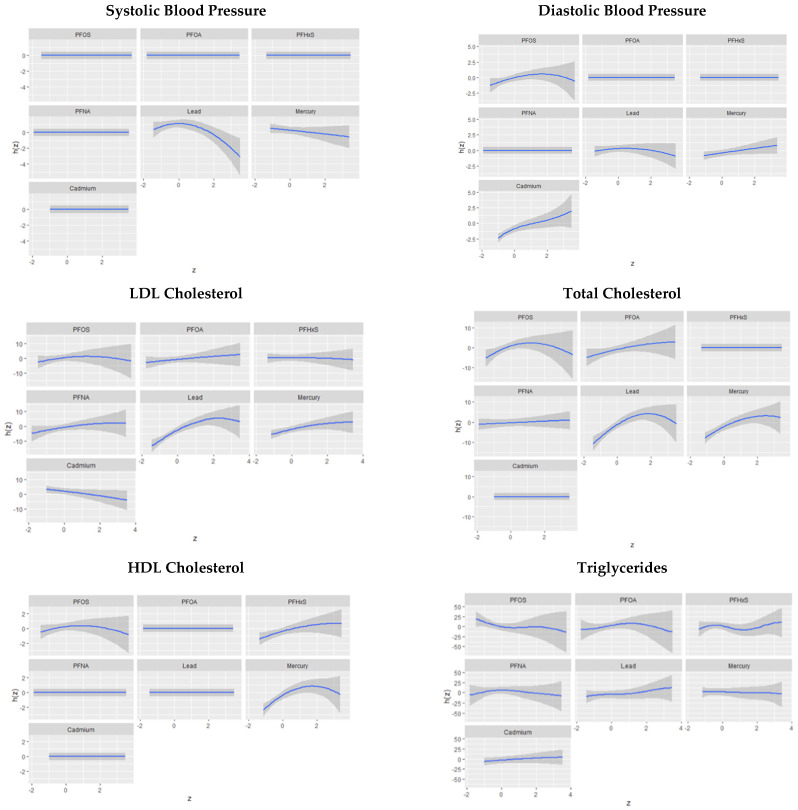
Univariate association of PFAS and metals on cardiovascular related outcomes.

The univariate approach visually examines the individual effect of PFOA, PFOS, PFNA, PFHxS, Hg, Cd, and Pb on cardiovascular disease risk outcomes. Figure 5 shows the impact of an individual PFAS and metals on cardiovascular-related outcomes when other PFASs and metals are fixed at the median. The grey bands represent 95% confidence intervals.

**Figure 6:**
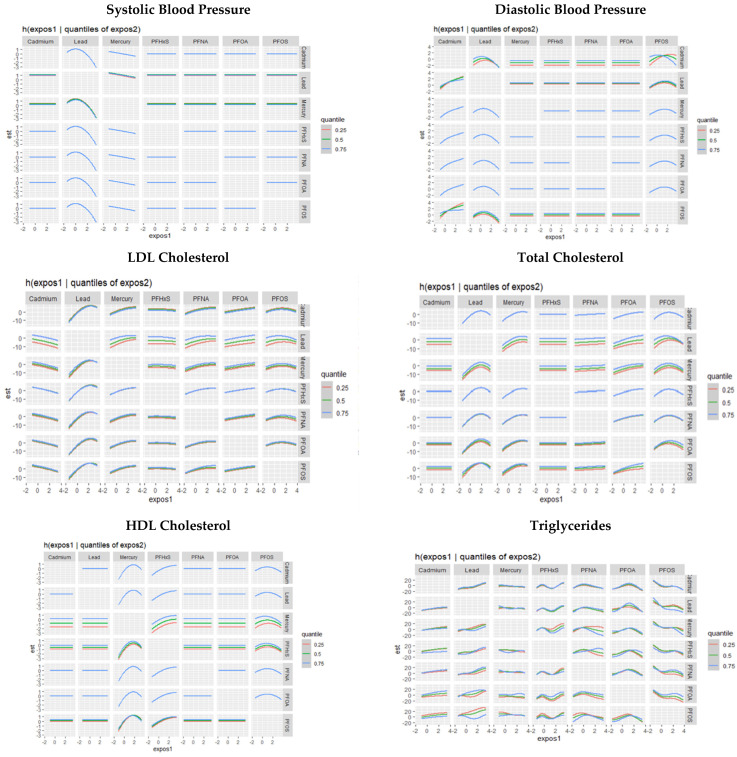
Univariate association of PFAS and metals on cardiovascular-related outcomes.

Figure 6 demonstrates bivariate associations of PFASs and metals with individual cardiovascular related markers.

#### 3.6.2. Single-Variable Effects of PFASs and Metals on Individual Cardiovascular-Related Markers

**Figure 7:**
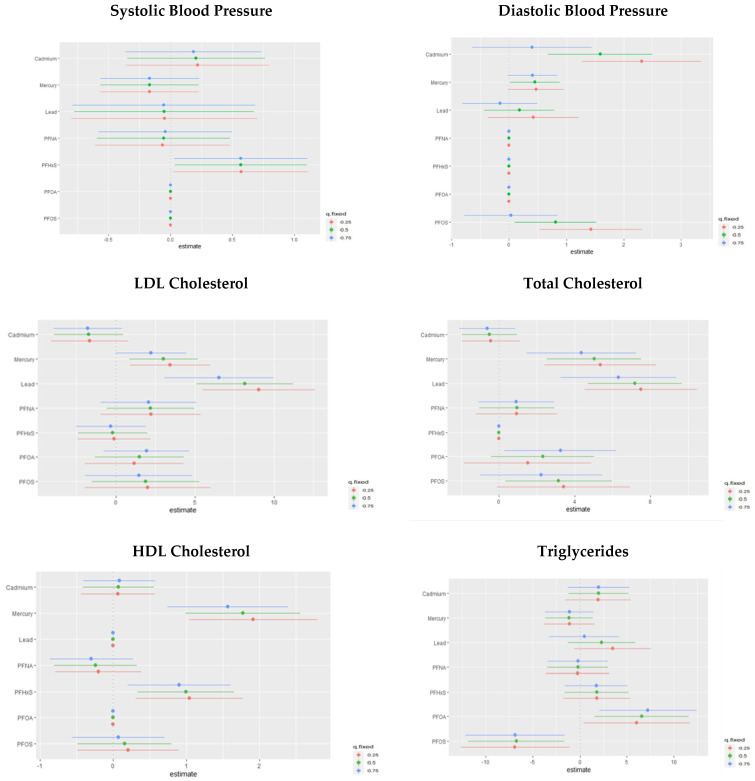
Chart showing the single-variable effect of PFASs and metals at increasing quantiles for cardiovascular-related markers.

The single-variable effect helps to understand the effect of a single predictor at different quantiles. Figure 7 demonstrates the single-variable effects of PFAS and metal components on individual CVD-related markers at 0.25, 0.5, and 0.75 quantiles. These figures focus on assessing the individual contributions of various exposure factors, namely PFASs and metals, to specific cardiovascular-related variables. This assessment of single-variable effects is instrumental in understanding the isolated influence of each environmental factor on distinct CVD-related outcomes. The results presented in these figures provide insights into how each exposure variable independently affects CVD markers.

### 3.7. Single-Variable Interaction Terms of PFASs and Metals on Individual CVD-Related Markers

**Figure 8:**
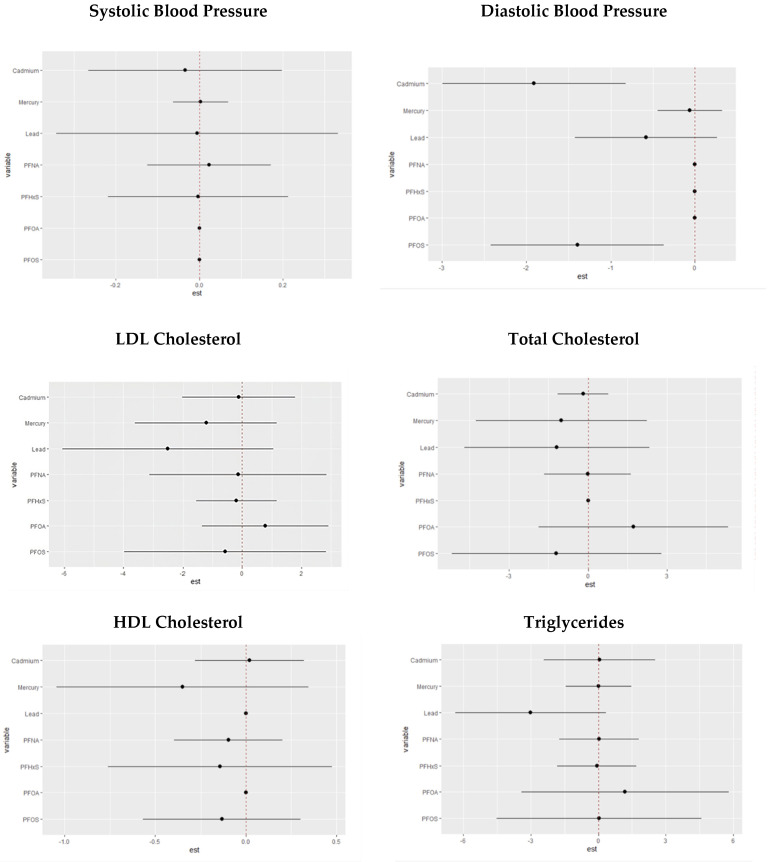
Chart showing the single-variable interaction terms of PFOA, PFOS, PFNA, PFNA, Hg, Cd, and Pb at increasing quantiles of other PFAS and metal variables for cardiovascular-related markers.

We explored the interaction effects between PFAS and metal exposures. Our analysis estimates the probability of the interaction inclusion for each pair of variables, indicating whether their joint effect significantly contributes to explaining the CVD outcome variable beyond their main effects. Figure 8 shows the overall effect of PFOA, PFOS, PFNA, PFNA, Hg, Cd, and Pb at increasing quantiles. The charts show the single variable effect when the other PFAS and metal components are fixed to their 25th quantile compared to when they are set at the 75th.

## 4. Discussion

The findings from this study provide valuable insights into the associations between environmental pollutants and CVD outcomes, shedding light on the impact of age and gender on these relationships. Age emerges as a consistent factor associated with adverse CVD outcomes, underscoring the importance of considering age in cardiovascular risk assessments. The relationship between age and cardiovascular disease was confirmed by Lakatta in his comprehensive review on the matter [41]. Indeed, age, though typically viewed as a non-modifiable risk factor, regrettably surpasses all modifiable factors, such as lipids, blood pressure, and smoking, in predicting clinical CVD events [42]. Nevertheless, the effects of age can be modified to reduce cardiovascular disease risk through considering understudied factors such as the environment.

In our study, sex-based disparities are evident in the levels of various pollutants, with men exhibiting higher concentrations of PFOA, PFOS, PFNA, PFHxS, mercury, and lead compared to women, while Cd levels were more elevated in women than men. These sex-specific variations emphasize the need for tailored risk assessment strategies based on sex. The difference noted for Cd may be based on sex steroids [43]. Indeed, Cd can serve as both an estrogen and an androgen within biological systems, and its capacity to bind to and activate steroid receptors is likely responsible for its hormone-like effects [44].

The study also reveals significant intercorrelations among PFAS variables, indicating a complex interplay within this class of chemicals. Linear regression analyses showed positive associations between specific pollutants and cardiovascular risk factors. Notably, PFOS exhibited a positive relationship with total cholesterol, while an interaction between PFOA and PFHxS was positively associated with LDL cholesterol. PFNA showed a significant association with triglycerides, while mercury showed a significant relationship with HDL cholesterol. Finally, Cd had a significant association with the OCBI. The associations of metals with CVD-related variables is well established [7,45]; in addition, PFASs have been associated with CVD risk, with platelet-related mechanisms having been implicated in the increase in cardiovascular events observed in populations with chronic exposure to PFAS [33]. These findings, though, take science further in that our study explored exposure to metals and PFASs concurrently. These findings thus speak to the effects of combined exposures on CVD risk.

We performed a Framingham Risk Score analysis, with our results highlighting that cadmium and lead levels were associated with increased CVD risk in both men and women. An interesting finding is the significant association between the interaction of PFOS, PFOA, and PFNA and CVD risk in females, underscoring the relevance of specific pollutant combinations in shaping cardiovascular risk. Combinations of PFASs are known to increase CVD risk [46], with factors such as exposure source and duration predicting the degree of CVD risk.

The BKMR analysis further explored the influence of individual pollutants on cardiovascular outcomes. We utilized Bayesian Kernel Machine Regression because it tends to outperform linear regression in multipollutant mixture modeling through accommodating non-linear relationships and interactions among predictors and outcomes. It provides flexibility in handling multicollinearity, performs variable selection, and offers uncertainty estimates, making it especially well suited for analyzing intricate exposure–response relationships. Furthermore, BKMR’s capability to integrate prior information and its resilience to outliers further enhance its utility in environmental health studies involving multiple exposures.

In our BKMR analysis, PFOS was identified as a significant factor affecting diastolic blood pressure, as indicated by a high PIP value. Lead demonstrated a high probability of influencing LDL cholesterol and total cholesterol. Mercury was also associated with total cholesterol. In conditional probability analysis, Cd and Pb exerted the most substantial impact on SBP and DBP. These findings suggest the differential effect of metals on different lipids and blood pressure, suggesting that combined exposure to multiple metals and PFAS may affect various processes in the development of cardiovascular-related diseases such as atherosclerosis. Atherosclerosis is a complex pathological process characterized by the accumulation of fatty plaques within arterial walls, leading to arterial narrowing and potential blockages. When individuals are exposed to a combination of different metals and PFAS, it appears it could influence various stages of this disease progression. This could involve a range of mechanisms, such as the promotion of inflammation, oxidative stress, endothelial dysfunction, and the alteration of lipid profiles, all of which are integral to the development and progression of atherosclerosis [47,48]. The interaction between metals and PFASs might act synergistically, potentiating each other’s effects or triggering cascades of events that exacerbate the disease process. This potential complex interplay underscores the importance of comprehensive risk assessment and the need to consider individual exposures and the cumulative impact of multiple environmental factors.

Univariate associations revealed that Pb was consistently and prominently linked to SBP and DBP when considered with other exposures. Research by Gu et al. also found that in multipollutant exposures, Pb tends to be the most prominent factor in CVD risk [49]. This may be due to its exceptionally long half-life, its strong prominent effect on the cardiovascular system, and its potential to combine with other contaminants and produce greater-than-additive effects. Total cholesterol exhibited significant relationships with Pb, Hg, PFOS, and PFOA. HDL cholesterol was influenced by mercury, PFHxS, and PFOS, while LDL cholesterol demonstrated a strong relationship with Pb and Hg. Finally, triglycerides were impacted by multiple variables, with PFOA being notably significant. These results collectively underscore the complex interplay between PFAS and metal exposures and lipid profiles and identify them as key determinants of cardiovascular health.

Bivariate associations emphasize the significance of Pb and its interactions across various cardiovascular outcomes. For SBP, Pb’s interactions with other pollutants are particularly significant. DBP, Pb, Cd, and PFOS maintained notable relationships. Total cholesterol was strongly associated with Pb, Hg, PFOS, and PFOA interactions, while HDL cholesterol demonstrated relationships with Hg, PFHxS, and PFOS interactions. In the context of LDL cholesterol, Pb’s interactions exhibited the strongest relationships, and for triglycerides, PFAS values showed stronger associations. These results again speak to Pb’s consistent significance across outcomes, especially in interactions with other pollutants, suggesting its substantial influence on CVD risk in the context of multipollutant exposure. Significant interventions to reduce the combined effects of metals and PFASs on CVD risk will likely need to begin through comprehensively addressing Pb contamination’s legacy.

In terms of single-variable effects on cardiovascular outcomes, Cd and PFHxS significantly impact SBP, while Cd and PFOS exert the largest influence on DBP. Total cholesterol was notably affected by Hg and Pb, with HDL cholesterol influenced by Hg and PFHxS. For LDL cholesterol, Pb and Hg demonstrated strong associations, with PFNA, PFOA, and PFOS also contributing. Triglycerides were significantly impacted by PFOA, with Cd, Hg, Pb, PFHxS, and PFNA also playing substantial roles. Identifying specific pollutants that considerably impact certain cardiovascular markers could allow for the development of targeted interventions and preventive measures. For instance, interventions aimed at reducing exposure to pollutants like Cd, PFHxS, and PFOS could positively affect blood pressure regulation.

The analysis of single pollutants interacting with other pollutants through comparing the 25th percentile to the 75th percentile revealed valuable insights. For SBP, the most significant impacts were attributed to changes in PFNA and mercury percentiles. For DBP, notable impacts arose from changes in PFNA, PFHxS, and PFOA percentiles. Total cholesterol was significantly affected by alterations in PFOA percentiles, and HDL cholesterol was influenced by changes in Cd, Pb, and PFOA percentiles. For LDL cholesterol, the most substantial impacts occurred with changes in PFOA percentiles, and PFOA also played a significant role in altering triglyceride levels. These findings underscore the intricate relationships between pollutants and their combined effects on cardiovascular outcomes, emphasizing the need for comprehensive risk assessment strategies that consider both individual and interactive pollutant contributions.

## 5. Conclusions

This study provides valuable insights into the intricate relationships between environmental pollutants and cardiovascular outcomes. It underscores the significance of age as a consistent and influential factor in predicting adverse cardiovascular events, highlighting the need to incorporate age into comprehensive risk assessments. Furthermore, gender-based disparities in pollutant levels emphasize the necessity for tailored risk assessment strategies based on sex, and the potential role of sex steroids in pollutant interactions adds depth to our understanding. Finally, the complex interplay among pollutants, particularly the combined exposure to metals and PFASs, underscores the need for holistic approaches to assess and mitigate the multifaceted risks associated with environmental contaminants in the context of cardiovascular health.

## Figures and Tables

**Table 1:** Overall Cardiovascular Biomarkers Index (OCBI).

Cardiovascular Biomarker	Biomarker Value Assigned Index Value (1)	Biomarker Value Assigned Index Value (0)
Diastolic blood pressure (DBP)	≤80	>80
Systolic blood pressure (SBP)	≤120	>120
High-density lipoprotein (HDL)	≥50 mg/dL	<50 mg/dL
Triglycerides (TG)	≤199 mg/dL	>199 mg/dL
C-reactive protein (CRP)	≤1 mg/dL	>1 mg/dL
Gamma glutamyl transferase (GGT)	≤40 U/L	>40 U/L
Total cholesterol (TC)	<200 mg/dL	>200 mg/dL
Glucose (FG)	<100 mg/dL	>100 mg/dL
Overall biomarker index	Sum of eight biomarker indicator values	

**Table 2:** Descriptive statistics for covariates.

Characteristics	
N	6237
Age: mean (SD)	43.0 (5.23)
Gender	
Female	51.3%
Male	48.7%
Education	
Less than high school	18.10%
High school or equivalent	21.35%
Some college/associate degree	30.41%
Bachelor’s degree or higher	30.13%
Race/ethnicity	
Black	10.84%
White	67.75%
Hispanic	14.16%
Other	7.25%
Household Income	
<25,000	31.72%
25,000–55,000	42.53%
55,000–75,000	19.72%
75,000+	6.02%
Marital Status	
Married or Living with a partner	62.8%
Single	37.2%

**Table 3:** Summary statistics (mean (SD)) of exposure variables and outcome variables by gender.

	Overall	Male	Female	*p* Value
N	6237	3100	3137	
PFOA	3.69 (1.32)	4.15 (3.20)	3.26 (2.38)	<0.0001
PFOS	12.84 (22.40)	15.35 (15.11)	10.46 (10.28)	<0.0001
PFNA	1.39 (0.48)	1.48 (1.50)	1.30 (1.20)	0.0000
PFHxS	2.5 (1.68)	3.13 (3.98)	1.92 (2.48)	<0.0001
Mercury	1.51 (1.79)	1.65 (1.99)	1.38 (3.08)	0.0067
Lead	1.47 (0.59)	1.72 (1.91)	1.23 (1.70)	<0.0001
Cadmium	0.48 (0.03)	0.45 (0.57)	0.51 (0.61)	0.0012
Systolic Blood Pressure	121.62 (17.69)	121.65 (15.10)	118.13 (18.03)	<0.0001
Diastolic Blood Pressure	69.05 (12.90)	70.24 (13.14)	68.00 (12.33)	<0.0001
Total Cholesterol	197.90 (42.80)	187.98 (42.41)	195.48 (41.90)	<0.0001
Triglycerides	132.08 (69.00)	140.99 (131.08)	115.49 (90.20)	0.0002
LDL cholesterol	118.8 (37.50)	113.17 (37.53)	115.17 (35.42)	0.2988
HDL Cholesterol	52.63 (15.58)	47.39 (13.22)	57.23 (16.26)	<0.0001
OCBI	5.7 (1.40)	4.71 (1.67)	5.11 (1.65)	<0.0001
Framingham Risk Score	9.24 (6.32)	8.08 (6.15)	10.34 (6.36)	<0.0001

**Table 4:** (**A**) Linear regression results for CVD risk on PFAS and metal exposures with interactions. Adjusted for age, BMI, gender, and taking prescription medication for hypertension and cholesterol. (**B**) Linear regression results for CVD risk on PFAS and metal exposures with interactions.

(**A**)								
**Stratum**	**Systolic Blood Pressure**		**Diastolic Blood Pressure**		**LDL Cholesterol**		**Total Cholesterol**	
**Term**	**Coefficient Estimates** **(95% CI)**	***p*-Value**	**Coefficient Estimates** **(95% CI)**	***p*-Value**	**Coefficient Estimates** **(95% CI)**	***p*-Value**	**Coefficient Estimates** **(95% CI)**	***p*-Value**
N	3513		3523		1747		3648	
Intercept	95.63 (3.99, 5.00)	<0.0001	54.57 (49.9, 59.23)	<0.0001	62.84 (49.9, 75.74)	<0.0001	139.48 (127.03, 151.73)	<0.0001
PFOS	–	–	–	–	–	–	–	–
PFNA	–	–	–	–	–	–	–	–
PFOA	–	–	–	–	–	–	–	–
PFHxS	−4.46 (−8.56, −0.37)	0.0338	–	–	–	–	–	–
Mercury	–	–	–	–	11.57 (5.31, 17.83)	0.0007	4.36 (4.74, 13.07)	0.0001
Cadmium	8.72 (2.22, 15.21)	0.0106	10.67 (3.56, 17.80)	0.0047	–	–	–	–
Lead	–	–	2.62 (0.24, 5.00)	0.0321	10.53 (3.83, 17.83)	0.0031	8.49 (4.37, 12.61)	0.0002
Age	0.32 (0.28, 0.36)	<0.0001	0.08 (0.52, 0.11)	<0.0001	0.29 (0.14, 0.46)	0.0006	0.42 (0.31, 0.54)	<0.0001
BMI	0.30 (0.24,−0.37)	<0.0001	0.26 (0.20, 0.31)	<0.0001	0.61 (0.33, 0.90)	0.0001	0.49 (0.25, 0.73)	0.0002
Gender Male	4.31 (3.16, 5.46)	<0.0001	3.37 (1.04, 3.69)	0.0010	–	–	−9.65 (−13.12, −6.19)	<0.0001
PFOS:PFOA	–	–	–	–	–	–	–	–
PFOS:PFNA	–	–	–	–	–	–	–	–
PFOA:PFNA	–	–	–	–	–	–	–	–
PFOS:PFHxS	0.38 (0.02, 0.75)	0.0398						
PFOA:PFHxS	–	–	–	–	3.19 (0.28, 6.10)	0.032	1.72 (0.03, 3.41)	0.045
PFNA:PFHxS	–	–	–	–	–	–	–	–
Mercury:Cadmium	–	–	–	–	–	–	–	–
Mercury:Lead	–	–	–	–	–	–	−3.43 (−6.41, −0.44)	0.026
Cadmium:Lead	−6.16 (−11.37, −0.94)	0.0225	−7.60 (−12.04, −2.81)	0.0030				
PFOS:PFOA:PFNA	–	–	–	–	–	–	–	–
PFOS:PFOA:PFHxS	–	–	−0.05 (−0.10, −0.00)	0.0465	–	–	–	–
PFOS:PFNA:PFHxS	–	–	–	–	–	–	–	–
PFOA:PFNA:PFHxS	–	–	–	–	–	–	–	–
Mercury:Cadmium:Lead	–	–	–	–	–	–	–	–
PFOS:PFOA:PFNA:PFHxS	–	–	–	–	–	–	–	–
${Adjusted\;R}^{2}$	0.27		0.00		0.15		0.13	
(**B**)								
**Stratum**	**HDL Cholesterol**		**Triglycerides**			**OCBI**		
**Term**	**OR** **(95% CI)**	***p*-Value**	**OR** **(95% CI)**		***p*-Value**	**OR** **(95% CI)**		***p*-Value**
N	3648		1747			3651		
Intercept	63.79 (58.58, 68.99)	<0.0001	40.60 (17.06, 64.13)		0.0013	7.25 (6.41, 7.91)		<0.0001
PFOS	–	–	−4.17 (−6.78, −1.57)		0.0026	–		–
PFNA	–	–	–		–	–		–
PFOA	–	–	–		–	–		–
PFHxS	3.7 (0.51, 6.93)	0.0246	–		–	–		–
Mercury	2.36 (0.91, 3.81)	0.0024	–		–	–		–
Cadmium	–	–	28.6 (5.92, 51.28)		0.0150	−1.12 (−1.78, −0.36)		0.0032
Lead	–	–	–		–	–		–
Age	0.04 (0.02, 0.07))	0.0013	0.61 (0.39, 0.83)		<0.0001	−0.01 (−0.02, −0.01)		<0.0001
BMI	−0.58 (−0.65, −0.52)	<0.0001	1.74 (1.34, 2.14)		<0.0001	−0.05 (−0.06, −0.05)		<0.0001
Gender Male	−8.65 (−9.83, −7.47)	<0.0001	–		–	−0.49 (−6.64, −0.32)		<0.0001
PFOS:PFOA	–	–	0.93 (0.31, 1.54)		0.0044	–		–
PFOS:PFNA	–	–	–		–	−0.18 (−0.42, 0.11)		0.0482
PFOA:PFNA	–	–	–		–	–		–
PFOS:PFHxS	–	–	1.33		0.0149			
PFOA:PFHxS	−1.52 (−2.72, −0.32)	0.0149	(0.28, 2.39)		–	–		–
PFNA:PFHxS	–	–	–		–	–		–
Mercury:Cadmium	–	–	–		–	0.84 (0.07, 1.53)		0.0250
Mercury:Lead	–	–	–		–	–		–
Cadmium:Lead	–	–						
PFOS:PFOA:PFNA	–	–	–		–	–		–
PFOS:PFOA:PFHxS	0.10 (0.00, 0.19)	0.0486	−0.38 (−0.64, −0.12)		0.0149	–		–
PFOS:PFNA:PFHxS	–	–	–		–	–		–
PFOA:PFNA:PFHxS	–	–	–		–	–		–
Mercury:Cadmium: Lead	–	–	–		–	–		–
PFOS:PFOA:PFNA:PFHxS	–	–	–		–	–		–
${Adjusted\;R}^{2}$	0.30		0.18			0.13		

**Table 5:** PFAS and metal association with the Framingham Risk Score.

Stratum	Male (N = 962)		Female (N = 741)	
Term	Coefficient Estimates (95% CI)	*p*-Value	Coefficient Estimates (95% CI)	*p*-Value
Intercept	–	–	−3.61 (−6.99, −0.25)	0.0362
PFOS	–	–	–	–
PFNA	–	–	–	–
PFOA	−0.70 (−2.60, −0.80)	0.0005	–	–
PFHxS	–	–	–	–
Mercury	–	–	–	–
Cadmium	3.02 (1.78, 4.25)	<0.0001	4.35 (2.40, 6.29)	<0.0001
Lead	1.56 (1.00, 2.12)	<0.0001	1.75 (1.00, 2.50)	<0.0001
BMI	0.25 (−0.65, −0.52)	<0.0001	0.21 (0.14, 0.27)	<0.0001
PFOS:PFOA	0.05 (0.01, 0.09)	0.0112	−0.07 (−0.13, −0.01)	0.0295
PFOS:PFNA	–	–	–	–
PFOA:PFNA	–	–	–	–
PFOS:PFHxS	–	–	–	–
PFOA:PFHxS	–	0.0149	0.47 (0.02, 0.92)	0.393
PFNA:PFHxS	–	–	–	–
Mercury:Cadmium	–	–	–	–
Mercury:Lead	–	–	–	–
Cadmium:Lead	–	–		
PFOS:PFOA:PFNA	–	–	0.04 (0.00, 0.07)	0.0689
PFOS:PFOA:PFHxS	–	–	–	–
PFOS:PFNA:PFHxS	–	–	–	–
PFOA:PFNA:PFHxS	–	–	–	–
Mercury:Cadmium: Lead	–	–	–	–
PFOS:PFOA:PFNA:PFHxS	–	–	–	–
${Adjusted\;R}^{2}$	0.15		0.20	
Adjusted for BMI				

**Table 6:** BKMR models for cardiovascular-related variables.

BKMR Models Showing PIP for Exposure to PFAS and Metal and by Health Outcome						
	Systolic Blood Pressure	Diastolic Blood Pressure	LDL Cholesterol	Total Cholesterol	HDL Cholesterol	Triglycerides
N	3515	3515	1690	3515	3515	1690
PFOS	0.0000	0.9548	0.4340	0.5724	0.0220	0.3026
PFOA	0.0000	0.0000	0.0790	0.3984	0.0000	0.4552
PFHxS	0.0062	0.0000	0.0298	0.0000	0.1038	0.0264
PFNA	0.0046	0.0000	0.3986	0.0240	0.0068	0.0392
Lead	0.1840	0.0000	0.9960	1.000	0.0000	0.1668
Mercury	0.0014	0.1044	0.2444	0.9736	0.9722	0.0222

The degree of blue color suggests the probability that PFAS is an influential factor affecting the Outcome Variable, with darker shades indicating a higher probability.

**Table 7:** Hierarchical BKMR results for diastolic blood pressure.

	Group	Group PIP	Cond PIP
PFOS	1	0.910	1.0000
PFOA	1	0.910	0.0000
PFHxS	1	0.910	0.0000
PFNA	1	0.910	0.0000
Lead	2	0.994	0.0805
Mercury	2	0.994	0.0000
Cadmium	2	0.994	0.9195

**Table 8:** Hierarchical BKMR results for systolic blood pressure.

	Group	Group PIP	Cond PIP
PFOS	1	0.050	0.0400
PFOA	1	0.050	0.1200
PFHxS	1	0.050	0.1600
PFNA	1	0.050	0.6800
Lead	2	0.368	0.9945
Mercury	2	0.368	0.0000
Cadmium	2	0.368	0.0054

**Table 9:** Hierarchical BKMR results for total cholesterol.

	Group	Group PIP	Cond PIP
PFOS	1	0.988	0.9089
PFOA	1	0.988	0.0162
PFHxS	1	0.988	0.0000
PFNA	1	0.988	0.0749
Lead	2	1.000	1.0000
Mercury	2	1.000	0.0000
Cadmium	2	1.000	0.0054
Notes			

**Table 10:** Hierarchical BKMR results for HDL cholesterol.

	Group	Group PIP	Cond PIP
PFOS	1	0.202	0.1782
PFOA	1	0.202	0.0000
PFHxS	1	0.202	0.8218
PFNA	1	0.202	0.0000
Lead	2	1.000	0.0000
Mercury	2	1.000	1.0000
Cadmium	2	1.000	0.0000
Notes			

**Table 11:** Hierarchical BKMR results for LDL Cholesterol.

	Group	Group PIP	Cond PIP
PFOS	1	0.962	0.0000
PFOA	1	0.962	0.0187
PFHxS	1	0.962	0.0000
PFNA	1	0.962	0.9812
Lead	2	1.000	1.0000
Mercury	2	1.000	0.0000
Cadmium	2	1.000	0.0000
Notes			

**Table 12:** Hierarchical BKMR results for triglyceride cholesterol.

Hierarchical BKMR Results for Triglycerides			
	Group	Group PIP	Cond PIP
PFOS	1	0.410	0.0000
PFOA	1	0.410	0.6390
PFHxS	1	0.410	0.0829
PFNA	1	0.410	0.2780
Lead	2	0.694	0.2478
Mercury	2	0.694	0.2075
Cadmium	2	0.694	0.5447
Notes			

## Data Availability

The NHANES dataset is publicly available online, accessible at cdc.gov/nchs/nhanes/index.htm (accessed on 2 October 2023).

## Appendix A. Framingham Risk Scor

**Criteria 1: Age by Gender.**		
**Female**		
**Framingham Risk Score by Gender**	**Age**	**Point**
Female	≥20 & ≤34	−7
	35 & ≤39	−3
	40 & ≤44	0
	45 & ≤49	3
	50 & ≤54	6
	55 & ≤59	8
	60 & ≤64	10
	65 & ≤69	12
	70 & ≤74	14
	75 & ≤79	16
**Male**		
Male	≥20 & ≤34	−9
	35 & ≤39	−4
	40 & ≤44	0
	45 & ≤49	3
	50 & ≤54	6
	55 & ≤59	8
	60 & ≤64	10
	65 & ≤69	11
	70 & ≤74	12
	75 & ≤79	13

**Criteria 2: Age and Total Cholesterol by Gender.**			
**Female**			
**Framingham Risk Score by Gender**	**Age**	**Total Cholesterol**	**Point**
Female	≥20 & ≤39	<160	0
	≥20 & ≤39	≥160 & <200	4
	≥20 & ≤39	≥200 & <240	8
	≥20 & ≤39	≥240 & <280	11
	≥20 & ≤39	≥280	13
	40 & ≤49	<160	0
	40 & ≤49	≥160 & <200	3
	40 & ≤49	≥200 & <240	6
	40 & ≤49	≥240 & <280	8
	40 & ≤49	≥280	10
	50 & ≤59	<160	0
	50 & ≤59	≥160 & <200	2
	50 & ≤59	≥200 & <240	4
	50 & ≤59	≥240 & <280	5
	50 & ≤59	≥280	7
	60 & ≤69	<160	0
	60 & ≤69	≥160 & <200	1
	60 & ≤69	≥200 & <240	2
	60 & ≤69	≥240 & <280	3
	60 & ≤69	≥280	4
	70 & ≤79	<160	0
	70 & ≤79	≥160 & <200	1
	70 & ≤79	≥200 & <240	2
	70 & ≤79	≥240 & <280	3
	70 & ≤79	≥280	4
**Male**			
**Framingham Risk Score by Gender**	**Age**	**Total Cholesterol**	**Point**
Male	≥20 & ≤39	<160	0
	≥20 & ≤39	≥160 & <200	4
	≥20 & ≤39	≥200 & <240	7
	≥20 & ≤39	≥240 & <280	9
	≥20 & ≤39	≥280	8
	40 & ≤49	< 160	0
	40 & ≤49	≥160 & <200	3
	40 & ≤49	≥200 & <240	5
	40 & ≤49	≥240 & <280	6
	40 & ≤49	≥280	8
	50 & ≤59	<160	0
	50 & ≤59	≥160 & <200	2
	50 & ≤59	≥200 & <240	3
	50 & ≤59	≥240 & <280	4
	50 & ≤59	≥280	5
	60 & ≤69	<160	0
	60 & ≤69	≥160 & <200	1
	60 & ≤69	≥200 & <240	1
	60 & ≤69	≥240 & <280	2
	60 & ≤69	≥280	3
	70 & ≤79	<160	0
	70 & ≤79	≥160 & <200	0
	70 & ≤79	≥200 & <240	0
	70 & ≤79	≥240 & <280	1
	70 & ≤79	≥280	1

**Criteria 3: Smoker.**			
**Female**			
**Framingham Risk Score by Gender**	**Smoker**	**Age**	**Point**
Female	Yes	20 & Age ≤ 39	9
	Yes	40 & Age ≤ 49	7
	Yes	50 & Age ≤ 59	4
	Yes	60 & Age ≤ 69	2
	Yes	70 & Age ≤ 79	1
	No		0
**Male**			
Male	Yes	20 & Age ≤ 39	8
	Yes	40 & Age ≤ 49	5
	Yes	50 & Age ≤ 59	3
	Yes	60 & Age ≤ 69	1
	Yes	70 & Age ≤ 79	1
	No		0

**Criteria 4: HDL.**		
**Female**		
**Framingham Risk Score by Gender**	**HDL**	**Point**
	HDL ≥ 60	−1
	HDL ≥ 50	0
	HDL ≥ 40	1
	HDL < 40	2
**Male**		
Male	HDL ≥ 60	−1
	HDL ≥ 50	0
	HDL ≥ 40	1
	HDL < 40	2

**Criteria 5: The participant has Systolic Blood Pressure and Treatment status.**			
**Female**			
**Framingham Risk Score by Gender**	**Treatment Status**	**Systolic Blood Pressure**	**Point**
Female	Treated	<120	0
	Treated	≥120 & <130	3
	Treated	130 & <140	4
	Treated	140 & <160	5
	Treated	≥160	6
	Untreated	<120	0
	Untreated	≥120 & <130	1
	Untreated	130 & <140	2
	Untreated	140 & <160	3
	Untreated	≥160	4
**Male**			
Male	Treated	<120	0
	Treated	≥120 & <130	1
	Treated	130 & <140	2
	Treated	140 & <160	2
	Treated	≥160	3
	Untreated	<120	0
	Untreated	≥120 & <130	0
	Untreated	130 & <140	2
	Untreated	140 & <160	2
	Untreated	≥160	3
Note: **Women:** The following point totals are related to different 10-year risks in %. Under 9 points: <1%; 9–12 points: 1%; 13–14 points: 2%; 15 points: 3%; 16 points: 4%; 17 points: 5%; 18 points: 6%; 19 points: 8%; 20 points: 11%; 21 = 14%, 22 = 17%, 23 = 22%, 24 = 27%, and >25= over 30%. **Men:** The following point totals are related to different 10-year risk in %. Less than 1 point: <1%; 1–4 points: 1%; 5–6 points: 2%; 7 points: 3%; 8 points: 4%; 9 points: 5%; 10 points: 6%; 11 points: 8%; 12 points: 10%; 13 points: 12%; 14 points: 16%; 15 points: 20%; 16 points: 25%; 17 points or more: over 30%.			
